# Event based surveillance of Middle East Respiratory Syndrome Coronavirus (MERS- CoV) in Bangladesh among pilgrims and travelers from the Middle East: An update for the period 2013–2016

**DOI:** 10.1371/journal.pone.0189914

**Published:** 2018-01-16

**Authors:** A. K. M. Muraduzzaman, Manjur Hossain Khan, Rezina Parveen, Sharmin Sultana, Ahmed Nawsher Alam, Arifa Akram, Mahmudur Rahman, Tahmina Shirin

**Affiliations:** 1 Deparment of Virology, Institute of Epidemiology, Disease Control & Research (IEDCR), Dhaka, Bangladesh; 2 Department of Pathology and Microbiology, Dhaka Dental College, Dhaka, Bangladesh; 3 Former Director, Institute of Epidemiology, Disease Control & Research (IEDCR), Dhaka, Bangladesh; Kliniken der Stadt Köln gGmbH, GERMANY

## Abstract

**Introduction:**

Every year around 150,000 pilgrims from Bangladesh perform Umrah and Hajj. Emergence and continuous reporting of MERS-CoV infection in Saudi Arabia emphasize the need for surveillance of MERS-CoV in returning pilgrims or travelers from the Middle East and capacity building of health care providers for disease containment. The Institute of Epidemiology, Disease Control & Research (IEDCR) under the Bangladesh Ministry of Health and Family welfare (MoHFW), is responsible for MERS-CoV screening of pilgrims/ travelers returning from the Middle East with respiratory illness as part of its outbreak investigation and surveillance activities.

**Methods:**

Bangladeshi travelers/pilgrims who returned from the Middle East and presented with fever and respiratory symptoms were studied over the period from October 2013 to June 2016. Patients with respiratory symptoms that fulfilled the WHO MERS-CoV case algorithm were tested for MERS-CoV and other respiratory tract viruses. Beside surveillance, case recognition training was conducted at multiple levels of health care facilities across the country in support of early detection and containment of the disease.

**Results:**

Eighty one suspected cases tested by real time PCR resulted in zero detection of MERS-CoV infection. Viral etiology detected in 29.6% of the cases was predominantly influenza A (H1N1 and H3N2), and influenza B infection (22%). Peak testing occurred mostly following the annual Hajj season.

**Conclusions:**

Respiratory tract infections in travelers/pilgrims returning to Bangladesh from the Middle East are mainly due to influenza A and influenza B. Though MERS-CoV was not detected in the 81 patients tested, continuous screening and surveillance are essential for early detection of MERS-CoV infection and other respiratory pathogens to prevent transmissions in hospital settings and within communities. Awareness building among healthcare providers will help identify suspected cases.

## Introduction

Middle East Respiratory Syndrome Coronavirus (MERS-CoV), a newly emerged novel corona virus, is associated with severe acute respiratory infection with high mortality rates [1]. MERS-CoV is an enveloped, single stranded positive sense RNA virus belonging to lineage C betacoronavirus. It was first isolated in the Kingdom of Saudi Arabia (KSA) from a patient with acute pneumonia who subsequently died of renal failure in September 2012 [2,3]. Although MERS-CoV was suspected primarily as zoonotic in origin butanimal to human transmission is not fully understood yet [4]. Human-to-human transmission has been documented in several clusters among health-care providers and contacts [5–8]. Since 2012, the World Health Organization (WHO) reported 1841 laboratory-confirmed MERS-CoV cases including 652 deaths from 28 countries[9]. All reported cases had an epidemiological link either directly or indirectly and around 80% of the cases were reported from KSA [10,11].The crude case fatality rate was 35% and males more than 60 years of age having underlying co-morbid condition are at higher risk of developing life threatening severe disease following MERS-CoV infection[9,12].

Millions of pilgrims from more than 180 countries across the globe unite in the holiest sites of KSA during the Hajj pilgrimage [13]. Elderly pilgrims with different underlying medical conditions and socioeconomic backgrounds from different parts of the world come in close contact during religious rituals in a closely defined area, most likely increasing susceptibility to respiratory tract infection[12,14,15]. More than 30% of pilgrims from Bangladesh are over 60 years of age [16]. Movement of these huge numbers of returning pilgrims might pose a potential risk of transmitting MERS-CoV in Bangladesh and other countries across the world. Taking all these into account, the International Health Regulations (IHR) Emergency Committee advised strengthening MERS-CoV surveillance capacities to ensure timely reporting of any identified cases in countries with returning pilgrims [17]. The Institute of Epidemiology, Disease Control & Research (IEDCR) under the Bangladesh Ministry of Health and Family Welfare (MoHFW) is mandated to lead outbreak investigations, surveillance and disease containment in the country, hence responsible for MERS-CoV screening in pilgrims/ travelers returning from the Middle East with respiratory illness (S1 Text).

A study conducted in the United Kingdom reported that respiratory infections among returning pilgrims were mainly caused by influenza and other respiratory viruses, such as Adeno virus, Respiratory syncytial virus, Human metapneumovirus and Para influenza viruses other than MERS-CoV [18]. To determine if this is the case for Bangladesh, this study was conducted to detect the viral pathogens responsible for respiratory infections among returning Bangladeshi pilgrims and other travelers from the Middle East.

## Materials and methods

### Patients

From October 2013 to June 2016 pilgrims and people returning from the Middle East presented with respiratory symptoms and having an epidemiological link were enrolled through active screening at the point of entry. After risk assessment by the medical team, suspected patients were admitted to the Kurmitola General Hospital isolation unit. In addition, travelers arriving with no clinical presentation received a health card mentioning the sign and symptoms of MERS-CoV infection. Self reported cases who developed symptoms within 14 days of arrival were included in sample collection along with admitted patients. The health care providers were instructed to use WHO standard Personal Protective Equipment (PPE) including N95 masks during handling of the patients.

### Samples

Upper respiratory tract (URT) nasal and throat swabs and lower respiratory tract (LRT) sputum were collected from 81suspected cases. Samples from self reported cases were collected in the sample collection room of IEDCR. Samples from hospitalized patients, samples were transported to the IEDCR Virology laboratory, using category A shipping regulations, in accordance with the WHO guidance on the Regulation for the transport of infectious agents 2013–14 [19].

### Respiratory virus screening

Nucleic acid was extracted and purified from samples according to manufacturer’s instruction (Viral RNA/DNA mini kit, PureLink, life technologies, CA, USA). Real time qRT-PCR assays for detection of MERS-CoV RNA was performed by using TaqMan technology. Primers and probes for upstream of the envelope (UpE) and non-structural protein 2 (N2) gene were used, provided by Centers for Disease Control (CDC), USA (CDC, catalog # KT0136). The sensitivity and specificity of the RT-PCR assay kit is 100% [20]. In addition RT-PCR was done for detection of nucleic acid of other respiratory viruses such asinfluenza A with subtype, influenza B, respiratory syncytial virus (RSV), parainfluenza virus types 1–4, human metapneumovirus, and adenoviruses [18]. Primers and probes for theserespiratory viruses were also supplied by CDC, Atlanta, USA. Ribonucleoprotein (RP) was used as internal control.

### Ethical statement

The samples were collected from the patients for laboratory investigation includingdetection of MERS-CoV, who had respiratory illness and epidemiological link as an emergency response where ethical clearance is exempted. Informed written consent was taken from all patients or patient’s guardian in case of minor prior to sample collection for MERS-CoV testing.

## Results

### Patients

Depending upon the clinical manifestations and epidemiological link, 81 cases were included in the study (S1 Data). Of them 45 were male and 36 were female, with age ranging from 14 months to 81 years (mean age: 49 ± 14 years) (Table 1).

**Table 1:** Demographic characteristics of 81 patients tested, October 2013 to June 2016.

	0–16 years	17–65 years	>65 years	Total (%)
Male	0	41	4	45 (55.6%)
Female	1	33	2	36 (44.4%)
Total	1 (1.2%)	74 (91.4%)	6 (7.4%)	81 (100%)

### Respiratory virus detection

**Fig 1:**
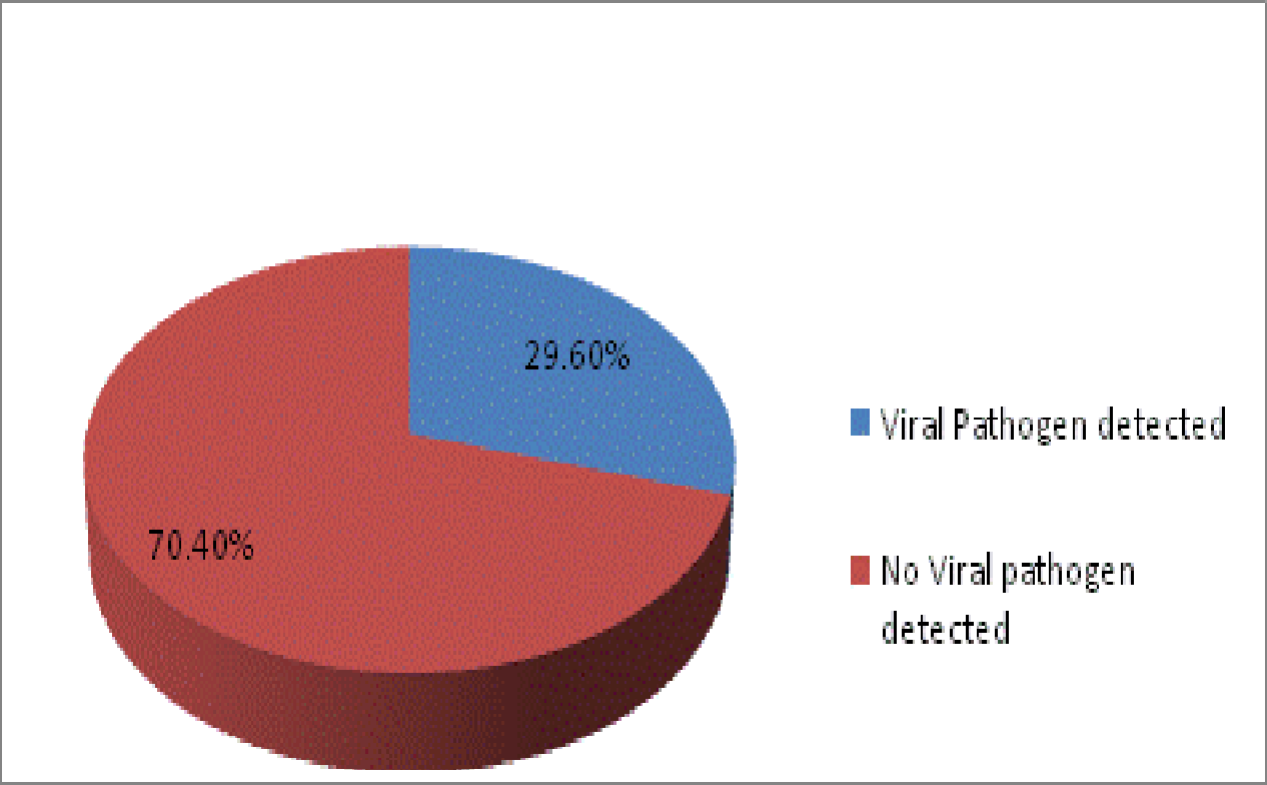
Total number of patients with a viral pathogen.

**Fig 2:**
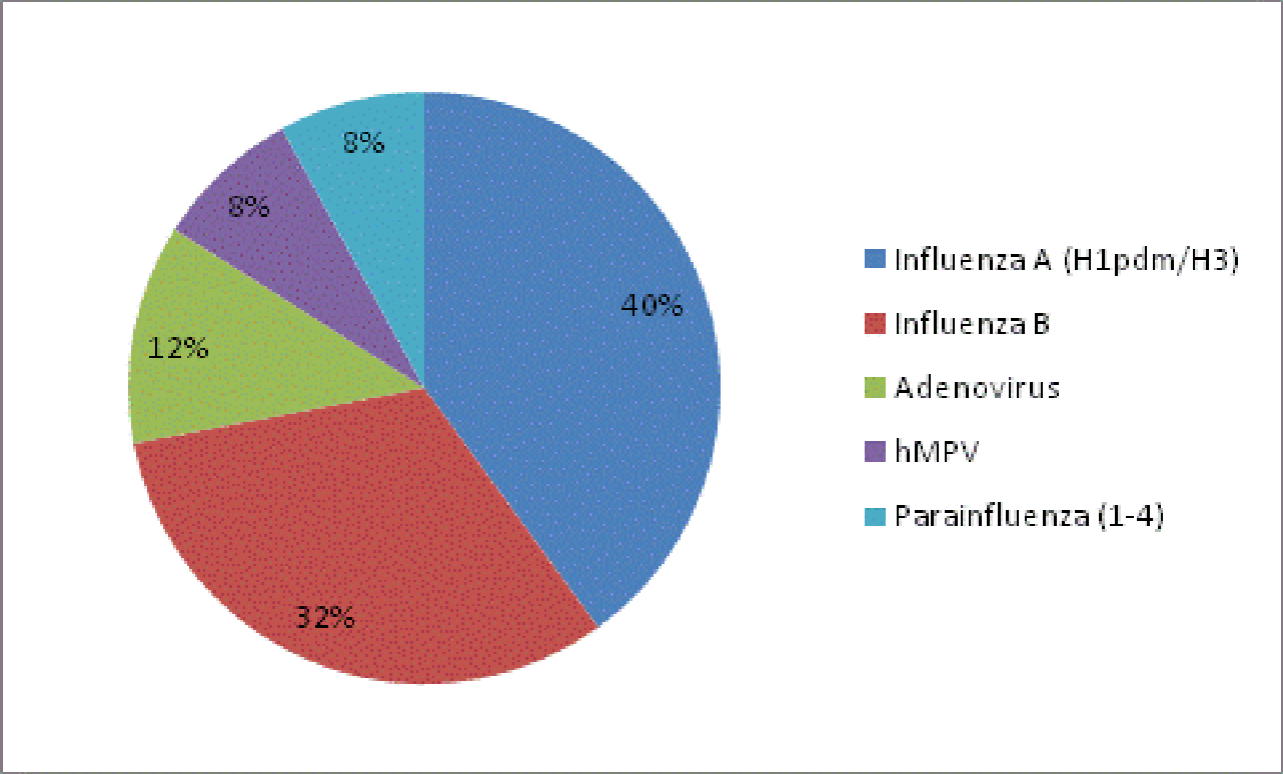
Respiratory viral pathogens detected in pilgrims and returning travelers.

**Fig 3:**
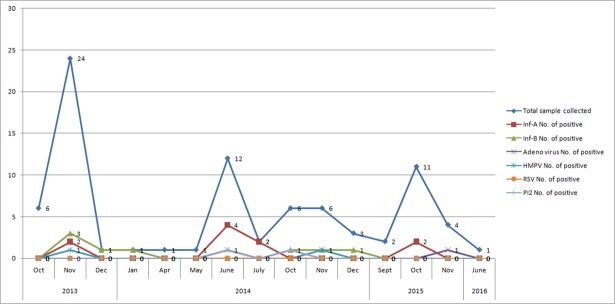
Testing of respiratory viral pathogens in 2013 to 2016 by month.

Of all the samples obtained from the 81 patients tested, none was positive for MERS-CoV. However, a viral etiology for clinical illness was found in 29.6% of cases (Fig 1). Among these positive samples, almost equal number of patients were found positive for influenza A (n = 10; either H1N1 or H3N2) and influenza B (n = 8). Additional respiratory pathogens detected were Adenovirus (2, 8%), human metapneumovirus (2, 8%) and parainfluenza 1–3 (2, 8%) (Fig 2).The highest number of samples were tested in October 2013 post-Hajj period (n = 24). During 2014 and 2015 the number of samples in the same time period was about half compared to 2013, 12 and 11 respectively. Up to June’2016 only one suspected case was tested and found negative for viral respiratory pathogens (Fig 3).

## Discussion

The emergence of various respiratory pathogens at different time points for instance, Severe Acute Respiratory Syndrome Corona virus (SARS-CoV) in 2003 in China, H1N1 influenza pandemic in 2009, and the emergence of another novel corona virus, MERS-CoV in the Kingdom of Saudi Arabia (KSA) in 2012, has engendered potential public health threats across the world [3,21,22].

The laboratory findings of this study of returning pilgrims from KSA and other travellers with symptoms of respiratory illness were found negative for MERS-CoV infection, but around 22% of them were infected by either influenza A or influenza B virus. Similar findings were observed among pilgrims from France in 2013[23]. The data from National Influenza Surveillance Bangladesh (NISB) revealed that, in the last five years the overall prevalence of influenza virus infection is 11%. Data also shows that the influenza season in Bangladesh starts in April and ends in September with peak in June-July [24]. However, the seasonality of influenza infection in KSA is different, beginning in September and peaking in November-December[25]. During this study period the Hajj was in the month of September and October. In the last four years pilgrims from Bangladesh received influenza vaccine recommended for Southern Hemisphere, as Northern Hemisphere vaccine is not available during that time. It is documented that influenza infection among vaccinated pilgrims is very common and may be due to mismatch strains[26]. This hypothesis, thus supports the higher prevalence of influenza infection among vaccinated Bangladeshi pilgrims in this study.

There was no evidence of MERS-CoV carriage among pilgrims from Bangladesh, suggesting no events of acquisition of MERS-CoV infection during the Hajj with other pilgrims and/ or contact with local people. Despite the small sample size compared to the overall Hajj pilgrim population (>1.9 million), lack of nasal carriage of MERS-CoV is evident, indicates that chance of viral transmission is low [27].

The challenges for laboratory diagnosis of MERS-CoV largely depend on the quality and type of respiratory tract specimens received[28]. Moreover, MERS-CoV causes a wide spectrum of clinical presentations in disease severity (mild to severe) which is associated with underlying co-morbid conditions [29]. Mild MERS-CoV infection may be missed among returning pilgrims if they recover spontaneously without attending any healthcare facilities. Thus, additional serological tests are required to determine the sero-conversion status to establish evidence of infection[30].

In order to detect and investigate any possible cases of MERS-CoV infection among returning pilgrims and travelers with epidemiological links, many countries, including Bangladesh, have established enhanced surveillance systems. Fortunately, none of the returning pilgrims were detected for MERS-CoV and few sporadic travel-associated MERS-CoV cases have been reported outside the Arabian Peninsula, mainly in Europe, North Africa, and Asia [18,31–33]. However few or no secondary cases were identified, with an exception in the Republic of Korea where the disease was spread to 185 cases from a single index case who had visited several countries in the Middle East[34].

To conclude, despite the lack of nasal carriage and lower transmission rate of this virus; higher incidence of morbidity and mortality and recent outbreak in South Korea with devastating outcome demands continuous monitoring and further investigations to ensure public health security.

## Supporting information

S1 Text Disease containment measures taken by IEDCR. (DOCX)

S1 Data Complete data file. (SAV)
