# Supplementary material for: Event based surveillance of Middle East Respiratory Syndrome Coronavirus (MERS- CoV) in Bangladesh among pilgrims and travelers from the Middle East: An update for the period 2013–2016
Source: PLoS One. 2018 Jan 16;13(1):e0189914. doi: 10.1371/journal.pone.0189914 (PMC5770030; doi:10.1371/journal.pone.0189914)
Supplement: S1 Text — (DOCX) [file pone.0189914.s001.docx]

**Disease containment measure:**

**Active screening at point of entry**

For containment of MERS-CoV infection active screening by thermal scanner was established at the port of entry for detection of suspected cases.

**Establishment of isolation unit**

A well equipped isolation unit was prepared at Kurmitola General Hospital, a Government hospital in Dhaka, capital of Bangladesh for management of the suspected MERS-CoV infected cases.

**Awareness building**

Information regarding MERS-CoV infection was disseminated through mass media for general population. Health card containing information about symptoms of MERS-CoV infection, instructions regarding possible measures and contact numbers of assign persons, to whom they will inform if the criteria fulfil, were distributed on arrival at the port of entry by MoH&FW, Bangladesh.

**Strengthening capacity**

A country wide training program was conducted among 3253 participants from primary, secondary and tertiary level health care facilities, on infection prevention and control, case identification, sample collection and transportation of sample to the reference laboratory at IEDCR as well as sensitization of the medical personnel at management level. Among these participants 8, 64, 1653, 1296, 152 and 80 were divisional Directors, Civil surgeons, physicians, nursing staffs, medical technologists (laboratory) and other health officials respectively.
